# Successful Transplantation of Frog Skin upon a Granulating Wound on the Foot

**Published:** 1887-04

**Authors:** 


### *SUCCESSFUL TRANSPLANTATION OF FROG SKIN UPON A GRANULATING WOUND ON THE FOOT.*

Dubousquet-Labordeni publishes in the *Gazette des Hôpitaux* the following clinical report: A young man twenty years of age received a severe burn of the foot from contact with molten iron. When the eschars were removed, the wound granulated nicely but showed no signs of cicatrization. On the dorsum of the foot the denuded surface extended backward from the little toe nine centimetres, and was four centimetres wide. On the plantar surface the skin was destroyed from the great toe backward eleven centimetres, and was six centimetres wide.

*It must be borne in mind that when modern French writers use the term "grain," which, fortunately, they seldom do, they mean five centigrammes, while the English grain is equal to about six and one-half centigrammes.

In order to compare grafts of human skin with those taken from the frog, four grafts were taken from the patient and placed upon the dorsal wound and four pieces of frog skin the size of the thumb nail were placed upon the plantar wound. Twenty-four hours afterward the dressing was removed and one of the grafts on each surface was detached, but the other six adhered to the wound. For six days the frog skin grafts retained their color and then began to lose their pigment and assume the appearance of human skin. In three weeks, the plantar wound was diminished in size by a fourth; the dorsal wound had made less progress. In a month, the plantar wound was completely healed but the dorsal wound was still open. The cicatrix of the plantar wound was soft and pliable; that of the dorsal wound was harder, thicker and somewhat painful.

The author recommends the adoption of the following precautions in skin grafting: The wound should be well granulated, avoid all flow of blood and suppuration by the use of antiseptic dressings, maintain complete immobility of the parts for at least twenty-four hours after the operation.

The following is his method of operation: The wound is washed with a carbolized solution and thoroughly dried with absorbent cotton. Small pieces of frog skin previously dipped two or three times in a weak solution of carbolic acid are then rapidly placed upon the granulations. Pieces of blotting paper smaller than the grafts are placed upon the pieces of frog skin in order to obtain compression and absorb moisture, and over this use the ordinary Lister dressing.

